# CMR real-time, free-breathing, phase contrast flow quantification: a novel approach to assess ventricular coupling in constrictive pericarditis

**DOI:** 10.1186/1532-429X-13-S1-O33

**Published:** 2011-02-02

**Authors:** Paaladinesh Thavendiranathan, David Verhaert, Michael Walls, Sanjay Rajagopalan, Chung Yiu-Cho, Orlando Simonetti, Subha V Raman

**Affiliations:** 1 The Ohio State University, Columbus, OH, USA

## Background/objective

Constrictive pericarditis (CP) is an important cause of heart failure; however, with accurate diagnosis and directed treatment it is potentially curable. Cardiac magnetic resonance imaging (CMR) has played a diagnostic role, primarily by allowing assessment of pericardial morphology but with limited depiction of physiological changes. We sought to examine the feasibility of a novel CMR approach that enables real-time phase contrast (RT-PC) assessment of discordant respirophasic changes in trans-mitral and tricuspid flow velocity - the signature findings in CP - due to enhanced ventricular interdependence.

## Method

**Figure 1:**
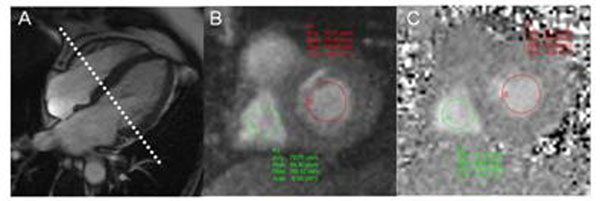
(A) Horizontal long axis cine image used for selection of RT-PC imaging plane. (B) Magnitude and (C) phase images obtained with RT-PC acquisition. Regions of interest for mitral inflow (red) and tricuspid inflow (green) are illustrated in both the magnitude and phase images.

**Figure 2:**
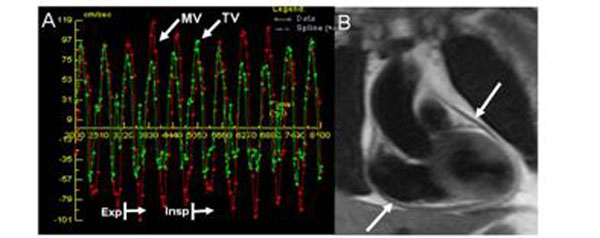
(A) RT-PC trans-mitral and tricuspid flow illustrating significant resiprophasic variation. (B) Dark blood turbo spin echo image illustrating pericardial thickening (arrows).

Patients referred to the CMR lab pre-pericardectomy or for assessment of suspected CP were included. Following routine CMR examination for CP, transmitral (MV) and tricuspid valve (TV) flow velocities were simultaneously obtained by through-plane RT-PC imaging during unrestricted respiration using a slice position to include both valves (Figure 1) with the following parameters: TR/TE=13.7ms/2.5ms, water excitation pulse with flip angle=25^o^, 10mm slice thickness, 160x120 matrix, EPI factor=15, TSENSE rate=2, slice thickness=10mm, and VENC=150cm/s. Shared velocity encoding was used to achieve an effective temporal resolution of 55ms and typically, 200-400 phases were obtained. The diagnosis of CP was confirmed using a combination of clinical history, diagnostic imaging, invasive hemodynamic measurements, intra-operative findings, and histopathology. Regions of interest at the mid-portion of the MV and TV were chosen on the PC images (Figure 1). Peak velocity data from average of 4 neighboring pixels for both valves were displayed simultaneously (Figure 2). The percentage change in velocity were calculated for MV as (MV expiratory E velocity - inspiratory E velocity)/(inspiratory E velocity) and for TV as (TV inspiratory E velocity - expiratory E velocity )/( expiratory E velocity).

## Results

**Figure 3:**
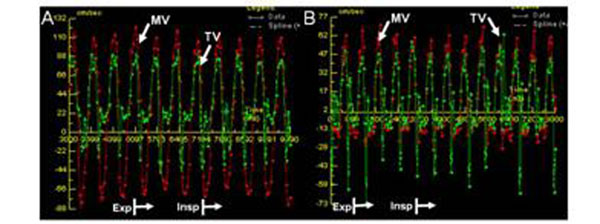
(A)RT-PC flow post pericardectomy in the same patient as Figure 2 illustrating absence of significant respirophasic variation. (B) RR-PC trans-mitral and tricuspid flow in a healthy volunteer illustrating lack of significant respirophasic variation.

9 patients (7 men, age 56±17 years) and 9 healthy volunteers (6 men, age 31±10) were included. All patients had increased pericardial thickness (6.3±1.5mm), a respirophasic shift of the interventricular septum, and inferior vena cava enlargement. Discordant respirophasic flow velocities across the mitral and tricuspid valves were recorded in all CP patients (Figure 2), with mean trans-mitral and tricuspid flow velocity variation measuring 46±21% and 60±16% respectively, compared to 17±5% (p=0.003) and 30±13% in controls (p<0.001) (Figure 3).

## Conclusions

Reciprocal respirophasic changes in mitral and tricuspid inflow velocity in CP can be simultaneously displayed by RT-PC imaging. This provides essential hemodynamic information, which in conjunction with other morphological and functional changes is a useful addition to the diagnostic armamentarium of CMR for the diagnosis of CP.

